# Prevalence of goitre and associated factors among school-aged children in Guraferda District, Southwest Ethiopia

**DOI:** 10.3389/fpubh.2025.1546149

**Published:** 2025-03-10

**Authors:** Gemechis Tuke, Walelign Mengistu, Kidist Kuratu, Sebsibe Elias, Miesa Gelchu

**Affiliations:** ^1^School of Public Health, Institute of Health, Bule Hora University, Bule Hora, Ethiopia; ^2^Public Health Department, Mizan-Aman College of Health Science, Mizan-Aman, Ethiopia

**Keywords:** child, goitre, iodine deficiency, associated factors, prevalence, cross-sectional study, Ethiopia

## Abstract

**Introduction:**

Goitre is a significant public health problem, particularly in underdeveloped countries like Ethiopia. Iodine Deficiency Disease is the leading cause of preventable brain injury in children, resulting in poor academic performance.

**Objectives:**

To determine the prevalence of goitre and associated factors among children aged 6–12 years in Guraferda District, Southwest Ethiopia in 2024.

**Methods:**

A community cross-sectional survey was conducted with 949 children ages 6–12 years who lived in selected kebeles in Guraferda District. Structured questionnaires, physical examinations, and iodized salt tests were all used to collect data. To identify factors related with goitre, a logistic regression analysis was performed using SPSS version 26. Statistical significance was determined at *p* < 0.05 using adjusted odds ratios and 95% confidence intervals.

**Results:**

The prevalence of Goitre among school-age children in this study was 37.6, 95% CI: 34.4, 40.8%. Female gender (AOR = 1.614, 95% CI: 1.199, 2.172), mothers with non-formal education (AOR = 1.93, 95% CI: 1.437, 2.592) (AOR = 1.93, 95% CI: 1.44, 2.592), rural residence (AOR = 2.291, 95% CI: 1.162, 3.239), storing salt near heat sources (AOR = 1.407, 95% CI: 1.042, 1.900), low food diversification status (AOR = 4.928, 95% CI: 3.332, 7.289), and consuming cabbage at least once a week (AOR = 2.874, 95% CI: 2.012, 4.106) were positively associated with Goitre, while consuming milk at least once a week (AOR = 0.217, CI: 0.145, 0.324) was negatively associated with Goitre.

**Conclusion:**

The study findings indicate a high prevalence of Goitre in the area. Factors such as being female, living in rural areas, mothers with no formal education, storing salt near heat sources, consuming cabbage, and low food diversification were associated with increased odds of Goitre. Therefore, it is recommended to ensure universal access to iodized salt and raise awareness in the community about the importance of using iodized salt.

## Introduction

Goitre, characterized by an unusual swelling of the thyroid gland, is among the most prevalent endocrine issues affecting children and adolescents (1). It represents one of the most severe outcomes of prolonged iodine deficiency in the human body, particularly in children residing in areas with low iodine levels (2). The condition is typically triggered by a significant intake of goitrogenic foods such as millet, sweet potatoes, corn, and cabbage, along with a minimal consumption of iodine-rich foods (2, 3). Iodine rich foods like Seaweed (nori, kelp, kombu, wakame) Fish, shellfish (cod, canned tuna, oysters, shrimp) are not commonly consumed in this study area (4).

The diagnosis of goitre depends on the visibility of the thyroid gland and the degree of its enlargement or the presence of nodules inside it. As a result, in 1979, it was advised that the palpation technique be employed as the most precise and reliable way for detecting endemic goitre and grading its severity (5). Iodine deficiency is caused by either insufficient iodine consumption in the diet or the intake of goitrogens (6). A total goitre rate (TGR) of 5% or higher is suggested as the threshold to signify a public health issue according to the consensus reached by major international organizations, including the WHO (7). Goitre classified based on WHO/UNICEF/ICCIDD classification scheme as follows: Grade 0: None or no goitre (palpable or visible) Grade 1 or palpable: A goitre that is palpable but not visible when the neck is in the normal position, (i.e., the thyroid is not visibly enlarged). Grade 2 or visible: A swelling in the neck that is clearly visible when the neck is in a normal position and is consistent with an enlarged thyroid when the neck is palpated. The total goiter rate (TGR) is the percentage of people in a population who have a goiter, which is an enlarged thyroid gland. TGR is calculated by dividing the number of people with a goiter by the total number of people examined (8).

In 2020, globally, 21 countries still have insufficient iodine in their diets (9). The most recent global estimate estimates that 1.88 billion individuals, including 241 million school-age children, do not get enough dietary iodine (10). According to the WHO, as of 2004, 42.6% of Africa’s population suffers from low iodine intake (11). Over a 10-year period from 1993 to 2003, trend analyses showed that the prevalence of Goitre rose from 15.6 to 28.3% (12).

Furthermore, half of the world’s 736 million extremely poor people live in only five countries: India, Nigeria, the Democratic Republic of the Congo, Ethiopia, and Bangladesh. India (93% HHIS, mUIC in women of reproductive age 178 μg/L), Nigeria (93% HHIS, mUIC in SAC 130 μg/L), Democratic Republic of Congo (82% HHIS, mUIC in SAC 249 μg/L), and Ethiopia (86% HHIS, mUIC in SAC 104 μg/L) are all iodine-sufficient at the national level (9).

The prevalence of goitre among children aged 6–12 years varies according to numerous research conducted around the world. It was recorded as 20.5% in India, 11.4% in Rajasthan, 32% in Portugal, 24.2% in Iran, 22.3% in southern Sudan (12–16). In Ethiopia a systematic review reported a pooled prevalence of goitre among school-age children was 42.9% (95% CI: 38.8–46.9). The highest prevalence of goitre (46.7%) was observed in Oromia region and the lowest (26.3%) was observed in Benishangul-Gumuz region (17).

Ethiopia began its iodized salt program in the 1990s, and the government has achieved tremendous success in iodized salt coverage, resulting in sharply enhanced iodine intake across the country. The country has achieved and sustained greater than 89% Household (HH) iodized salt coverage from its lowest point of 15% coverage and subsequently improved iodine intake across its population (18). Based on the most recent available median urinary iodine concentration (UIC) data from 194 WHO Member States Ethiopia has adequate Iodine intake (19). In addition, iodine levels in salt surpassing 15 ppm were found in 42.7% of families. Additionally, 23.2% of households had salt levels that met the national threshold of 20–40 ppm (20). Despite the Ethiopian government’s efforts to implement mandated salt iodization over the past decade, an iodine shortage persists in the country (21). The prevalence of iodine deficiency among school age children, with mean urinary iodine concentration below the cut-off, was 48% (22). Systematic review and meta-analysis done reported the pooled prevalence of iodine deficiency among school-age children in Ethiopia was found to be 58% (95%CI 44.00–77.00) (23). Furthermore, several places have reported endemic cases linked to goitrogens present in drinking water, which may include specific chemical components that affect the synthesis of thyroid hormones (24). Goitrogens are chemicals that are toxic to the thyroid or that break down to produce toxic chemicals. Goitrogens are present in various foods, such as cassava, cabbage, turnips, and rutabagas. Cassava, for example, is a staple in Africa (25). Goitrogens such as fluoride, found in water, and thiocyanates, found in cassava and cruciferous vegetables, can interfere with iodine uptake. Foods are considered goitrogenic if they contain substances that inhibit thyroid function or iodine utilization (26).

The aim of this study is to assess the prevalence of goitre and associated factors among school age children (6–12 years of age) in Guraferda District, Southwest Ethiopia.

## Materials and methods

### Study design, setting and population

A cross-sectional study based in the community was carried out from February 1 to March 30, 2024, in Guraferda District, located in the Bench-Sheko Zone, which comprises one of the six Districts in this zone. The total population in the study area was estimated to be 51,016 (25,253 males and 25,763 females) according to the 2007 Gregorian calendar (G.C) Census conducted by the Central Statistics Agency (CSA), with a projected 10,411 households in 2024. Guraferda District is geographically located between 6°51′0″ north and 35°4′0″ east of the equator. It covers an area of 2,565.40 km^2^ and is at an elevation of 501–2,500 m above sea level. The mean annual temperature in the zone is 20–32°C, with an average rainfall of 700–1,500 mm. Guraferda District is made up of four urban kebeles (It is the smallest administrative unit in Ethiopia) and 27 rural kebeles, totalling 31 kebeles. The district contains five high schools, 48 primary schools, and three government-operated kindergartens alongside three private ones. Data from the Guraferda District Education office indicates that there are 9,307 school-age children residing in the district. The primary agricultural products produced in the district include maize, rice, millet, cassava, cabbage, godere (is a staple food in all Kebeles of the Guraferda District which was source of most of the daily food intake for large rural populations), honey, and various animal products. The study included all school-aged children (6–12 years) living in the area, excluding only children in the same age group with neck swelling unrelated to Goitre.

### Sample size determination and sampling procedures

#### Sample size determination

In order to establish the sample size for this study, a literature review was conducted for both objectives. After calculating the sample sizes for each objective individually, the larger sample size was selected for this study. This study has two specific objectives as follows:

1. To determine the prevalence of goitre and
2. To identify factors associated factors with Goitre.

##### The sample size determination for the 1st objective

The sample size for determining the prevalence of goitre was calculated using a single population proportion formula, based on the following assumptions: a confidence level of 95%, a margin of error of 5%, and a goitre prevalence of 28.37% (27) among school-age children.


$$n=\frac{{\left(Z1-\frac{\propto }{2}\right)}^{2}*P\left(1-p\right)}{d^{2}}$$

Where, n = sample size, Z *α*/2 = Critical value = 1.96, P = prevalence of goitre (28.37%), *δ* = precision (marginal error) = 0.05.

Then, $n=\frac{{\left(1.96\right)}^{2}\left(0.2837\right)\left(1-0.2837\right)}{0.04^{2}}≅488\text{,}$ by taking design effect 1.5andadding 10% non-response rate the total sample size will be = $\left(\left(488*1.5\right)+\left(\frac{488*1.5*10}{100}\right)\right)=805$ .

Finally, the sample size for the 1st objective was 805.

##### The sample size determination for the 2nd objective

The sample size for factors associated with goitre in school-aged children (6–12 years) was calculated from different studies (28, 29), using EPI info version 7.1 with a double population proportion formula to calculate the OR of the factors (Table 1). The assumptions were: 80% Power, 95% confidence level (z) and Ratio exposed of unexposed is 1:1.

**Table 1:** Sample size for the second objective.

S. no.	Factors	AOR	% Outcome among unexposed	Sample size	Design effect (1.5)	Non response rate (10%)	Final sample size
1	Using non iodized salt	2.20	18.28	302	302*1.5 = 453	46	499
2	Cabbage consumption	2.52	4.9	602	602*1.5 = 903	91	994
3	Living with family in a single room	2.30	42.26	204	204*1.5 = 306	31	337

Finally, the sample size obtained was the largest sample size from the two objectives, which was 994.

### Sampling procedures

**Figure 1:**
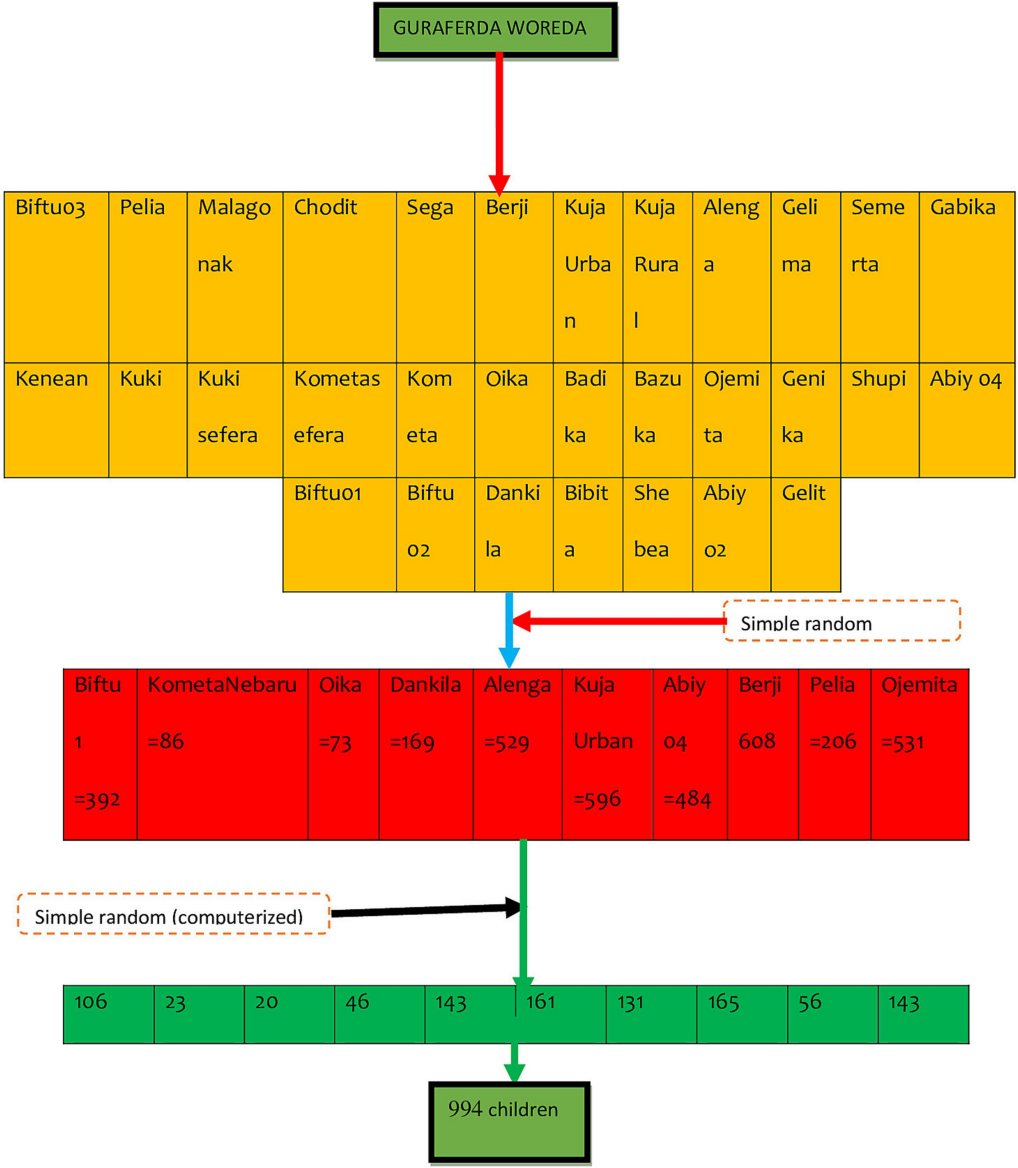
Schematic representation of sampling procedure of goitre prevalence and its associated factors among school-age children (6-12 years) in Guraferda District, Southwest, Ethiopia, 2024.

Guraferda District comprises a total of 31 kebeles. A simple random sampling method, specifically a lottery system, was used to select 30% of these kebeles. A survey was carried out in the chosen kebeles to assess the total number of school-age children living there. The overall sample was allocated proportionally among the selected kebeles. Households containing at least one school-age child were identified using family folder codes, which were then compiled to establish a sample frame. These codes were entered into Microsoft Excel, where random numbers were generated to select households. In cases where there were multiple school-age children (ages 6–12) in a selected household, one child was randomly chosen using a lottery method. If the household head was not present during data collection, a follow-up visit was arranged for the next day. If the household head remained absent after the second visit, a third visit was planned for the subsequent day. If the household head is still not available after three visits, the next household with at least one school-age child would be included in the study (Figure 1).

### Variables

#### Outcome variable

Goitre (presence or absence).

#### Explanatory variables

After a thorough review of the literature regarding factors that affect goitre prevalence, the study incorporated seventeen (17) explanatory variables identified in prior research. These variables included child age, child gender, parental educational background, parental occupations, marital status, household wealth level, source of water, place of residence, family size, rooms in the house, types of food consumed, types of salt used, dietary diversification, family history of goitre, and knowledge about goitre and its prevention (3, 28–32).

#### Data collection tools and procedures

Data were gathered through structured questionnaires administered by interviewers, physical examinations, and a quick iodized salt test. The questionnaires were adapted from existing literature (3, 27–33) and translated into the local language (Amharic) by a certified translator. The translation process followed a forward-backward translation method to ensure accuracy order to reflect the context of study area. Initially, the questionnaires were created in Amharic, then translated into English, and subsequently translated back into Amharic to ensure consistency. To verify the questionnaires’ accuracy, a pretest was carried out with 5% of the participants in the study. Physical examination for determining the status of goitre among school children, the participants were instructed to bring a handful of salt used by their home. The iodine content of the sample salt was tested by rapid iodized salt test kit. The salt sample was taken in a teaspoon and then a drop of the test solution was poured on the salt.

##### Physical/thyroid examination

The presence of goitre was evaluated through physical assessments of school-aged children (ages 6–12) and categorized using the WHO goitre staging system into grade 0, grade 1, or grade 2. Goitre was classified as absent (grade 0) if there was neither palpable nor visible swelling, as grade 1 when it was palpable but not visible, and as grade 2 when it was visible on the neck. Ultimately, goitre was deemed present if a child exhibited either grade 1 or grade 2 goitre, or both (8, 34).

##### The iodine content of the salt

In order to assess the presence of adequately iodized salt among the households sampled, the interviewer requested each household to provide a teaspoon (Approximately 5 g) of the salt used for food preparation the night before. The iodine content of the salt sample was measured using a rapid iodized salt testing kit (MBI test kit; MBI Kits International, Tamil Nagar, India). This kit included a stabilized starch-based solution that induces a chemical reaction observable through a color change. The salt sample was then placed in a small cup and spread out flat, as instructed with the testing kit. Two drops of the test solution from the white ampule were added onto the salt’s surface, and the resulting color was compared against a color chart within 1 min to gauge the iodine concentration (intense color). If there was no color change observed on the salt after a minute, additional test solution was applied to a new sample, adding up to five drops of recheck solution from the red ampule, followed by two drops of the test solution on the same spot for comparison with the color chart. Ultimately, the results were classified as either 0 ppm (no iodine in the salt), <15 ppm (light blue and insufficient iodine), or ≥15 ppm (deep blue and sufficient iodine content). This assessment utilized the Improved Iodized Salt Field Test Kit, Batch No. 014 MF FEB. 2020, EXP NOV. 2022, specifically for salt fortified solely with potassium iodide. An unopened ampule was utilized, and the kit was validated for visual detection of potassium iodide concentration with a detection limit of 15 ppm, yielding reliable results. The test kit was sourced from UNICEF via the Guraferda District Health Office. In the analysis, households showing iodine levels below 15 ppm, as well as those with no detectable iodine, were deemed inadequate, while households with iodine concentrations exceeding 15 ppm were classified as adequate, in accordance with findings from a previous study (35).

##### Household wealth status

Assets of the household were gathered through structured questionnaires modified from the 2019 Ethiopian District Health Survey (EDHS). The resulting data underwent analysis using Principal Component Analysis (PCA). Ultimately, Household wealth status was classified into quintiles based on a composite score derived from ownership of assets, housing characteristics, and access to services, as per the Ethiopian Demographic and Health Survey (EDHS) methodology in the order of Lowest, second, middle, fourth and highest (36).

##### Dietary diversification

To evaluate the dietary diversity levels, information about the food items consumed within the last 24 h by school-age children (ages 6–12) was gathered using a dietary diversity questionnaire tailored from criteria for measuring household and individual dietary diversity. A list of commonly consumed local food items was compiled by consulting key informants in the kebele. These food items were subsequently categorized into common food groups. School-age children from the surveyed households were interviewed regarding the foods they had eaten in the preceding 24 h using the 24-h food recall method. The food items ingested in that timeframe were classified into 12 distinct food groups. Ultimately, the data was divided into categories reflecting low dietary diversity (≤3 food groups), medium dietary diversity (4 and 5 food groups), and high dietary diversity (≥6 food groups) (37).

### Data quality control

To ensure the quality of the data, the supervisor and data collector participated in a two-day training focused on tools and methodologies. A pretest of the instrument was administered to 5% of samples from kebeles that were excluded from the study. A standardized checklist, adapted from guidelines for assessing household and individual dietary diversity, was utilized (37). The reliability of the instruments was assessed by measuring both intra-observer and inter-observer agreement using the Kappa coefficient. A Kappa coefficient value of 0.75 or higher is deemed satisfactory. For this evaluation, 20 questionnaires were used for both inter-observer and intra-observer reliability, resulting in a Kappa coefficient of 0.86, which satisfied the acceptable standards. During the data collection period, the supervisor and principal investigators reviewed each questionnaire every morning to confirm completeness. Furthermore, to guarantee the validity and reliability of goitre assessment, two data collectors examined each school-age child. In instances where the first two examiners disagreed on goitre ascertainment, a third examiner was consulted to evaluate, and the two consistent findings were utilized for diagnosis.

### Data management and analysis

The data was carefully examined and cleaned to address any missing or anomalous values. Data entry and analysis were performed using Epi Data version 4.6 and SPSS version 26.0, respectively. Descriptive statistics were computed for various variables as necessary. To assess the household wealth index, Principal Component Analysis (PCA) was employed, ensuring all prerequisites were satisfied. Given that the outcome variable was categorical, both binary and multivariable logistic regression analyses were utilized to control for potential confounding factors and to identify associations with the outcome variable. The independent variables in the logistic regression model were included through Stepwise multiple regression using forward selection. The Hosmer & Lemeshow Goodness of Fit Test was conducted to evaluate the appropriateness of the variables in predicting the dependent variables, resulting in a value of 0.795. Binary logistic regression was performed utilizing simple Logistic Regression where contributions to the model were considered significant at *p* < 0.25. All independent variables that showed *p* < 0.25 in bivariate analyses were incorporated into the multivariate model. Diagnostics for multi-collinearity indicated collinearity between cabbage and cassava consumption, with Variance Inflation Factor (VIF) values of 34.28 and 34.67, respectively. Because cassava consumption exhibited the highest VIF value, it was excluded from the model. Ultimately, only variables with a VIF <10 among the independent variables were retained in the model, with minimum and maximum VIF values of 1.01 and 1.60, respectively. The Adjusted Odds Ratio with a 95% Confidence Interval was reported, with the significance level established at *p* < 0.05. The findings were presented using text, frequency, and statistical summaries.

### Ethical approval

The study was carried out after receiving ethical approval with reference number of #I/O/H/I/R/B/024/14 from Bule Hora University’s Institutional Review Board. Permission was also obtained from the town administration office in Mizan-Aman. All study participants were provided with brief explanations about the purpose and benefits of the study. Written consent were obtained from parents or guardians in exchange for their full cooperation. Names and other personal information that could compromise the confidentiality of the respondents were not used. The confidentiality and privacy of the participants’ information were protected, and their right to withdraw or not participate was respected. Feedback forms were prepared for participants, providing an overview of the results from the physical measurements. Finally, those with goitre were referred to the nearest health facility

## Results

### Socio-demographic and economic characteristics

Among the 994 expected school-age children, 949, accompanied by their mothers or caregivers, took part in the study, achieving a response rate of 95.47%. The average age of the participants was 8.08 years (SD = 1.696). Regarding their place of residence, 687 (72.4%) were living in rural areas. Most of the children, 678 (71.4%), came from families consisting of five members or more, while 274 (28.9%) fell into the second quintile of wealth status. Nearly two-thirds (590, 62.2%) of the mothers/caregivers were engaged in farming occupations, and 477 (50.26%) as well as over half, 523 (55.11%), of the children’s mothers and fathers lacked formal education, respectively (Table 2).

**Table 2:** Socio-demographic characteristics of school-age children and their parents/caregivers in Guraferda District, Southwest Ethiopia, 2024 (*n* = 949).

Variables	Categories	Frequency	Percentage
Sex of child	Male	454	47.8
	Female	495	52.2
Age of child	6–8 years	704	74.2
	9–12 years	245	25.8
Religion of the mother’s/caregivers	Muslim	232	24.4
	Orthodox	328	34.6
	Protestant	376	39.6
	Others	13	1.4
Residence	Urban	262	27.6
	Rural	687	72.4
Marital status	Married	817	86.1
	Divorced	85	9.0
	Widowed	47	5.0
Mother’s educational level	Cannot read and write	221	23.3
	Read and write	256	27.0
	Primary	380	40.0
	Secondary	54	5.7
	College/University	38	4.0
Father’s educational leve	Cannot read and write	223	23.5
	Read and write	300	31.6
	Primary	351	37.0
	Secondary	51	5.4
	College/University	24	2.5
Mother’s occupation	House wife	129	13.6
	Farmer	590	62.2
	Government employee	61	6.4
	Private employee	39	4.1
	Merchant	130	13.7
Father occupation	Farmer	606	63.9
	Government employee	114	12.0
	Private employee	15	1.6
	Merchant	214	22.6
Households wealth status in quintile	Lowest	179	18.9
	Second	274	28.9
	Middle	166	17.5
	Fourth	179	18.9
	Highest	151	15.9
Family size	<5 family	271	28.6
	5 and above family	678	71.4

### Nutritional characteristics of school age children

**Figure 2:**
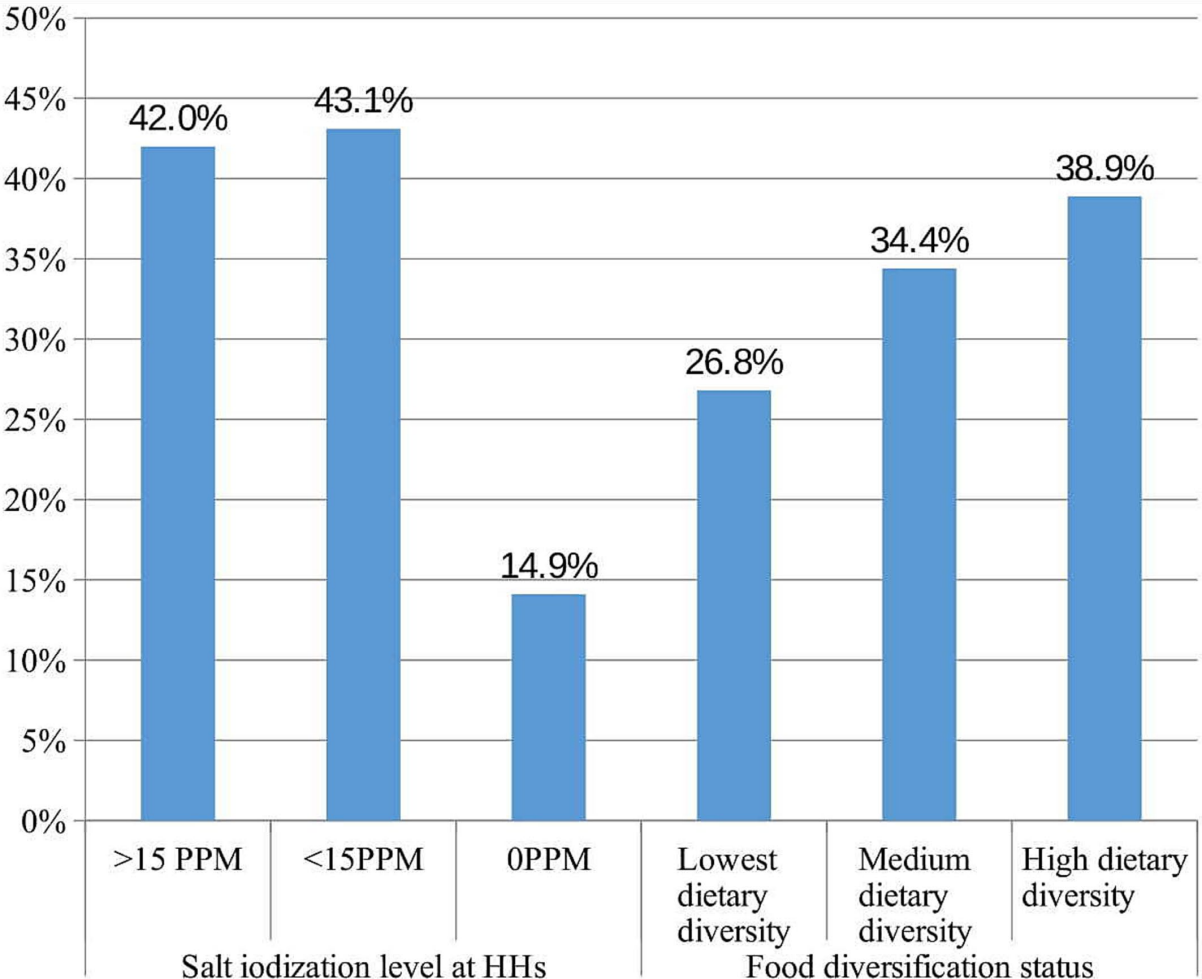
Nutritional characteristics of school age children in Guraferda District, Southwest Ethiopia, 2024.

The results from the rapid iodine test indicate that 141 (14.9, 95% CI: 12.6, 17.2%) of the household salt samples tested were non-iodized. Furthermore, 409 (43.1, 95% CI: 39.8, 46.3%) samples of household salt contained insufficient iodine levels (1–15 ppm), while only 399 (42, 95% CI: 38.9, 45.3%) exhibited adequate iodine levels (≥15 ppm). Regarding the dietary diversification status of children, 254 (26.8, 95% CI: 23.9, 29.5%) had the lowest dietary diversity, 326 (34.4, 95% CI: 31.4, 37.3%) had medium dietary diversity, and 369 (38.9, 95% CI: 35.9, 42.1%) achieved high dietary diversity (Figure 2).

### Dietary habit and goitrogenic food consumption by children

A significant majority of the children (82.8%) had regularly included eggs in their diet. Millet was the most frequently eaten goitrogenic staple, with 438 children (46.2%) reporting its consumption. Among the vegetables and dairy items, sweet potato and milk were consumed by 523 (55.1%) and 663 (69.9%) of the children, respectively (Table 3).

**Table 3:** Goitreogenic and non goitreogenic food feeding status of school age children (6–12 years) in Guraferda District, Southwest Ethiopia, 2024.

Variables	Categories	Frequency	Percent
Have you ever eaten cabbage	Never	522	55.0
	Once a week	77	8.1
	Two times a week	67	7.1
	3 and above times a week	283	29.8
	Never	522	55.0
Have you ever eaten cassava	Never	520	54.8
	Once a week	98	10.3
	Two times a week	148	15.6
	3 and above times a week	183	19.3
Have you ever eaten millet	Yes	438	46.2
	No	511	53.8
Have you ever eaten rice	Yes	793	83.6
	No	156	16.4
Have you ever eaten maize	Yes	433	45.6
	No	516	54.4
Have you ever eaten potato	Yes	523	55.1
	No	426	44.9
Have you ever eaten godere	Yes	792	83.5
	No	157	16.5
Have you ever eat acho	Yes	547	57.6
	No	402	42.4
Have you ever eat honey	Yes	566	59.6
	No	383	40.4
Have you ever drink milk	Yes	663	69.9
	No	286	30.1
Have you ever eat eggs	Yes	786	82.8
	No	163	17.2

### Knowledge and practice of caregiver toward goitre

Among the total caregivers/households surveyed, 665 (70.1%, CI: 67.3, 72.8%) demonstrated a good knowledge of goitre, whereas 284 (29.9%, CI: 27.2, 32.7%) displayed a poor knowledge. Furthermore, 384 (40.5%, CI: 37.4, 43.9%) of the caregivers indicated that they utilize unpacked salt (Table 4).

**Table 4:** Knowledge and practice of care giver towards goitre prevention in Guraferda District, Southwest Ethiopia, 2024.

Variables	Categories	Frequency	Percent
Level of knowledge about goitre in HH	Good knowledge	665	70.1
	Poor knowledge	284	29.9
Type of salt used in the HH	Un packed	384	40.5
	Packed	565	59.5
Place where you commonly buy salt	Open market	53	5.6
	Shops	896	94.4
Do you expose salt to sun light?	Yes	96	10.1
	No	853	89.9
Place of salt stored in the house?	Near to fire	398	41.9
	Away from fire	551	58.1
Type of salt container used in the HH	Storage without cover	349	36.8
	Storage with cover	600	63.2
When did you add iodized salt during cooking?	After cooking	98	10.3
	During cooking	851	89.7

HH, Household.

#### Prevalence of goitre

**Figure 3:**
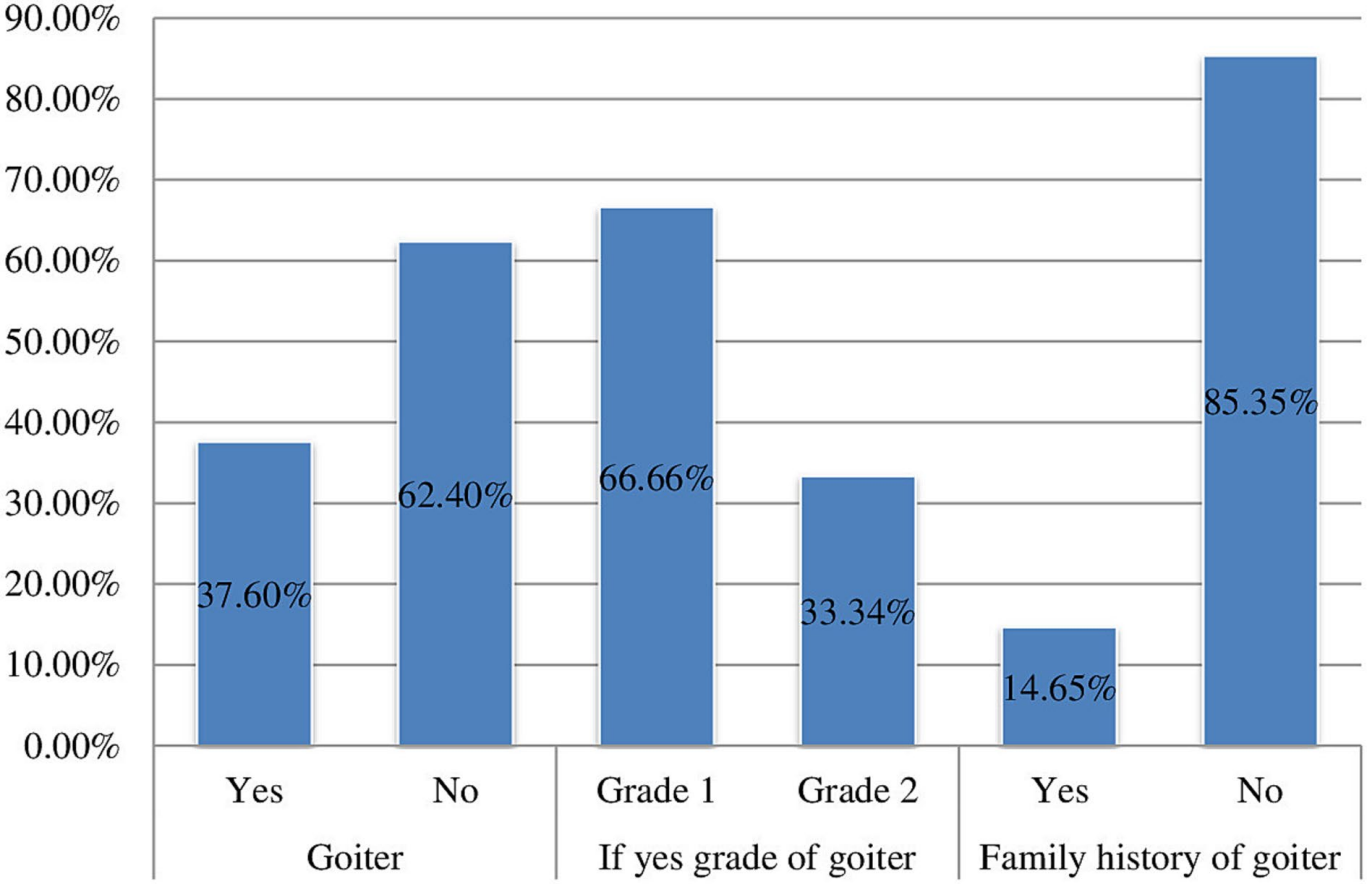
Prevalence of goitre among school age children (6–12 years) in Guraferda District, Southwest, Ethiopia, 2024.

The overall prevalence of goitre among children of school age in this research area was found to be 37.6% (95% CI: 34.8, 40.5%). Out of the total number of children with goitre, 238 (66.7, 95% CI: 61.2, 71.2%) presented with grade one goitre, while 119 (33.3, 95% CI: 28.8, 38.8%) had grade two. Furthermore, the prevalence of goitre was recorded at 36% for 6–8-year-olds and 42% for those aged 9–12 years. The occurrence of goitre was 44.2% among females and 30.04% among males. Among the participants in the study, 139 (14.6, 95% CI: 12.6, 16.9%) reported having a family history of goitre (Figure 3).

#### Factors associated with goitre

In the analysis using multivariable logistic regression, factors linked to goitre in school-age children included the child’s sex, dwelling location, maternal education level, consumption of milk, consumption of cabbage, keeping salt near heat sources, and the diversity of food intake (*p*-value <0.05). The likelihood of goitre occurrence was 1.578 times greater in female children compared to males (AOR = 1.578, 95% CI: 1.155, 2.157). Children whose mothers or caregivers had no formal education had nearly double the odds of developing goitre than those with mothers or caregivers who had formal education (AOR = 1.93, 95% CI: 1.44, 2.592). Moreover, children residing in rural areas had odds of goitre that were almost twice as high as those living in urban areas (AOR = 2.291, CI: 1.62, 3.24).

In addition, the odd of getting goitre was 1.4 times more likely in children whose families stored salt near heat than in those whose families kept salt away from heat sources (AOR = 1.41, 95% CI: 1.042, 1.90). Conversely, children with the lowest food diversification status faced a fivefold increase in goitre risk when compared to children with the highest food diversification status (AOR = 4.93, 95% CI: 3.332, 7.29). Likewise, children who consumed cabbage at least once weekly had nearly three times the odds of developing goitre compared to those who never ate cabbage (AOR = 2.874, 95% CI: 2.012, 4.106). Conversely, the chances of goitre were 78% lower in children who consumed milk at least weekly compared to those who did not consume milk (AOR = 0.217, CI: 0.145, 0.392) (Table 5).

**Table 5:** Multivariable analyses of factors associated with goitre among school age children (6–12 years) in Guraferda District, Southwest Ethiopia, 2024 (*N* = 949).

Variables	Goitre			*p* value	COR (95% C.I)	*p* value	AOR (95% C.I)
	Categories	Yes *N* (%)	No *N* (%)				
Child sex	Female	219 (44.24)	276 (55.56)	0.000	1.817 (1.391, 2.374)	0.002^*^	1.614 (1.199, 2.172)
	Male	138 (30.4)	316 (69.6)	1			
Maternal educational level	Non formal education	219 (45.9)	258 (54.1)	0.000	2.054 (1.572, 2.686)	0.001*	1.930 (1.437, 2.592)
	Formal education	138 (29.24)	334 (70.76)	1			
Residence of households	Rural	290 (42.2)	397 (57.8)	0.000	2.126 (1.550, 2.917)	0.001*	2.291 (1.620, 3.239)
	Urban	67 (25.6)	195 (74.4)	1			
Have you ever eat cabbage	Yes	149 (34.9)	278 (65.1)	0.117	1.236 (1.15, 1.611)	0.001*	2.874 (2.012, 4.106)
	No	208 (39.85)	314 (60.15)	1			
Have you ever eat milk	Yes	287 (43.9)	376 (56.1)	0.000	0.425 (0.311, 0.579)	0.001*	0.217 (0.145, 0.324)
	No	70 (24.5)	216 (75.5)	1			
Place of salt stored in the house?	Near to fire	161 (40.45)	237 (59.55)	0.126	1.230 (1.14, 1.61)	0.026*	1.407 (1.042, 1.900)
	Away from fire	196 (35.6)	355 (64.4)	1			
Food diversification status children	Lowest dietary diversification	145 (57)	109 (43)	0.000	4.302 (3.012, 6.4)	0.001*	4.928 (3.332, 7.289)
	Medium dietary diversification	77 (23.6)	249 (76.4)	0.000	2.306 (1.664, 3.2)	0.001*	2.540 (1.778, 3.629)
	Highest dietary diversification	135 (36.6)	234 (63.4)	1			

*indicates statistically significant variables at the *p*-value < 0.005.

## Discussion

Ethiopia has achieved and sustained greater than 89% Household (HH) iodized salt coverage from its lowest point of 15% coverage and subsequently improved iodine intake across its population (18). Despite the Ethiopian government’s efforts to implement mandated salt iodization over the past decade, an iodine shortage persists in the country (21).

The study revealed that 37.6% of school-aged children (ages 6–12) in the analyzed region were afflicted by goiter, designating it as an endemic area per the World Health Organization (WHO), which defines any region with a goiter prevalence exceeding 5% (8), which states that any area with a goitre prevalence rate greater than 5% is considered endemic. The finding corresponds with a research conducted in northwest Ethiopia involving children of the same age group and is also consistent with the national prevalence observed in studies undertaken in Debre Tabor town and Chole District (3, 27, 29, 30). However, the results of this study were lower than those from Anchar District, which found a goitre prevalence of 51.8%, Shebe Senbo District, which revealed a prevalence of 59.1%, and Northeast Ethiopia, which indicated a prevalence of 62.1% (28, 30, 32, 38). The variation in results can be explained by variations in annual precipitation and elevation across the study sites, which could lead to the loss of iodine-rich topsoil (39, 40).

The finding of this study is also lower than the study conducted in Butajira Town which reported a prevalence of 49.65% among pregnant women (41). The possible explanation for this difference might be attributed Iodine requirement will increase during pregnancy. Prevalence of Goitre in this study is much higher than a systematic review conducted in Ethiopia (42). This disparity could be explained by a systematic review study that pooled the prevalence of goitre from various studies, with individual prevalence ranging from 5 to 56.2%. The finding of this study is also lower than a systematic review and meta-analysis done in Ethiopia which reported the pooled prevalence of IDD to be 58% among school age children (23). The difference could be explained by the fact that the Iodine test method employed in our investigation differed from that used in the previously reported study.

The rate of goitre found among the school-aged children involved in this study was also greater than the prevalence noted in studies carried out in Pakistan and Iran which reported 35.0 and 24.2% (16, 43, 44). The greater incidence of goitre in children observed in our research, when compared to results from analogous studies, might be linked to the region’s mountainous terrain and the long-term poor soil conservation practices. This could have resulted in the depletion of the topsoil that is rich in iodine.

Millet was the most frequently eaten staple food in the study, with 438 children (46.2%) reporting its consumption. It is known for its goitrogenic effect (45) which might have increased the risk of developing goitre among study participants.

Female children had twice odds of developing goitre compared to male. This observation aligns with earlier research carried out in the Arsi Zone and North West Ethiopia (3, 28, 29). The increased occurrence of goitre in females could be attributed to the greater iodine needs of female children compared to their male counterparts, particularly at the onset of puberty (46). The results of our research oppose the findings of a study carried out in the Kohat District of Pakistan, which indicated that the prevalence of goitre was greater in males than in females (43). The differences could stem from genetic variations between the two populations being studied.

Children who ate cabbage at least once a week, and especially those who ate it more frequently, had three times the likelihood of developing goitre compared to those who had never eaten cabbage. This observation aligns with earlier research carried out in different areas of the country, including southwest Ethiopia, Chole District, and Arsi Zone (3, 29, 33). One potential reason for this might be that cabbage is a naturally occurring goitrogenic food with thiocyanate and isothiocyanate, which prevent the transport of iodine to the thyroid gland. This blocking of iodide transport in the body could result in the swelling of the thyroid gland (3).

Children who drank milk at least once a week were less likely to develop goitre than those who did not. Our results align with studies carried out in Chole District and Anchar District in Eastern Ethiopia (29, 32). This discovery aligns with information from multiple sources suggesting that milk offers beneficial nutrients that help prevent goitre (47). Although animal-based foods contain less iodine compared to plant-based foods, milk and dairy products are still considered a decent source of iodine (47).

The odd of developing goitre was five times greater in children who had the least diverse diets compared to those who had the most varied food options. This observation aligns with research carried out in the Amhara regional state, Adama city, and Bale, Ethiopia (48–51). One potential reason for this observation could be that a variety of foods facilitated better absorption of iodine by the body. Sufficient intake of dietary iodine is one approach to combat iodine deficiency disorders. Groups that primarily consume a uniform diet centered around cereals frequently experience shortages in iodine, along with other essential nutrients like vitamin A and iron (48, 52, 53).

Children aged 6–12 residing in rural regions had 2.29 times greater odds of developing goitre compared to their counterparts living in urban areas. This aligns with results from a survey conducted in Portugal and United states of America which indicated that endemic goitre predominantly impacts the rural population (15, 54, 55) The noted disparity could stem from the reality that individuals residing in rural regions might possess less awareness regarding the causes and prevention of goitre, along with restricted access to healthcare services in these communities. Additionally, challenges in obtaining salt when required may result in the rural population not utilizing iodized salt as effectively as those in urban areas. Moreover, factors such as sex, education level, place of birth, family income, site of residence, knowledge, dietary intake, unprotected water consumption, consumption of goitrogenic foods, and family history can affect the prevalence of goiter (3, 56, 57).

The research indicated that participants whose mothers or caregivers had non-formal education were twice as likely to develop goitre. Children whose mothers or caregivers possessed less education faced a greater risk of developing goitre compared to those with mothers who received formal education. Evidence from various areas of the country reinforces the finding that children of less educated mothers are more susceptible to developing goitre (27, 32). A study conducted in Khoramabad indicated that as the educational level of parents rises, the prevalence of goitre decreases (16). A potential reason for the finding mentioned is that mothers or caregivers without formal education might not be utilizing iodized salt correctly, resulting in improper practices.

Additionally, our research revealed that keeping salt close to a fire was notably linked to the occurrence of goitre. School-age children whose families or caregivers keep salt near a fire had 1.4 times greater odds of developing goitre compared to those whose families or caregivers store salt away from a fire. This result aligns with findings from Jimma Town, national-level study conducted in Ethiopia and Iran (30, 58, 59). One potential explanation for this observation could be that keeping salt close to a fire reduces the amount of iodization in salt at the household level (58, 60).

In this study, there was no association of iodine content with whether the salt was packed or unpacked, in contrast to earlier studies in Gondar, North West Ethiopia (61), Lalo Assabi District, West Ethiopia (16), and Robe town, South Central Ethiopia (62). Loss of iodine due to environmental factors from non-packaged salt was higher than from packaged salt in a previous study (63). The salt samples collected in this study were iodized using potassium iodate, rather than potassium iodide. As iodates are less soluble and more resistant to oxidation than iodide, the salt iodine content remains relatively constant under different environmental conditions (moisture, heat, and sunlight) even in unpacked salt (64, 65).

## Limitations of the study

In this study, the urinary iodine levels of the subjects were not tested due to resource constraints, which could have revealed their recent iodine intake status and helped with treatment and monitoring. Goitre was diagnosed using WHO goitre staging methods, but the gold standard diagnosis of goitre is ultrasound, which may result in misclassification of subclinical stages of cases. Using only a 24-h questionnaire (it does not represent individual variability and requires recall) (66).

## Conclusion

The prevalence of goitre in this study area was found to be 37.6%, classifying it as a goitre endemic area according to the World Health Organization (WHO), which states that any area with a goitre prevalence rate greater than 5% is considered endemic (8). Factors positively associated with goitre in this study included being female, mothers with no formal education, storing salt near fire, children with low food diversification, children living in rural areas, and children consuming cabbage at least once a week. We recommend that future studies in this region incorporate child growth and development measures to provide a more comprehensive understanding of the impact of IDD.

## Data Availability

The raw data supporting the conclusions of this article will be made available by the authors, without undue reservation.

## Abbreviations

AOR: Adjusted Odds Ratio
CI: Confidence Interval
PCA: Principal Component Analysis
COR: Crude Odds Ratio
CSA: Central Statistics Agency
DDS: Dietary Diversity Score
EDHS: Ethiopian Demographic Health Survey
EPHI: Ethiopian Public Health Institute
GP: General Practitioner
GC: Gregorian calendar
HH: Households
ICCIDD: International Council for the Control of Iodine Deficiency Disorders
IDD: iodine Deficiency Disorder
OR: Odds Ratio
PPM: Parts Per Million
SNNPR: South Nation and Nationalities of people Region
SAC: School age children
SPSS: Statistical Package for the Social Sciences
TGR: Total Goitre Rate
TSH: Thyroid Stimulating Hormone
UNICEF: United Nations International Children Emergency Fund
WHO: World Health Organization
